# Optimized production and characterization of a thermostable cellulase from *Streptomyces thermodiastaticus* strain

**DOI:** 10.1186/s13568-024-01787-0

**Published:** 2024-11-21

**Authors:** Mery S. Waheeb, Walid F. Elkhatib, Mahmoud A. Yassien, Nadia A. Hassouna

**Affiliations:** 1 Department of Microbiology and Immunology, Faculty of Pharmacy, Ain Shams University, Organization of African Unity St., POB: 11566, Abbassia, Cairo Egypt; 2 Department of Microbiology & Immunology, Faculty of Pharmacy, Galala University, New Galala City, Suez, Egypt

**Keywords:** Cellulase, *Streptomyces thermodiastaticus*, Optimization, Purification, Characterization

## Abstract

A high cellulase-producing bacterial isolate TS4 was recovered from an Egyptian soil sample and identified using 16S rRNA gene sequencing as *Streptomyces thermodiastaticus*. One-factor-at-a-time (OFAT) preliminary studies were carried out to determine the key factors affecting cellulase production by *S. thermodiastaticus* and their optimum ranges. The initial pH of the medium, carboxymethyl cellulose (CMC), tryptone, and NaCl concentrations were further optimized using a response surface Central Composite design. Fermentation under optimized variables of initial pH 6.0, presence of CMC, tryptone, and NaCl at concentrations of 2%, 0.03%, and 0.12%, respectively, resulted in 3.24 fold increase in cellulase productivity (2023 U/L) as compared to that under basal conditions (625 U/L). Cellulase production was also improved with a 4 Kilogray (KGy) dosage of gamma radiation. In comparison to the wild-type strain under basal circumstances, *S. thermodiastaticus* produced 5.1 fold more cellulase after a combination of model-based optimization and gamma radiation mutation. Cellulase was partially purified using ammonium sulfate precipitation followed by dialysis. The resulting cellulase was 1.74 times purified and its specific activity was 4.21 U/mg. The molecular weight of cellulase is 63 kDa as indicated by SDS-PAGE and zymogram. Its maximum activity was achieved at 60 °C and pH 5.0. In addition, it showed outstanding thermo-tolerance as it could retain its full activity after a 12-h incubation at 90 °C.

## Introduction

Cellulase is regarded as one of the most crucial enzymes on the industrial scale. About half of the dry weight of plant biomass is made up of cellulose, the substrate for cellulase. It is regarded as one of the most important carbon sources on the earth, with an annual biosynthesis rate of 100 billion tons by both plants and marine algae (Kumar et al. 2019). The cellulase enzyme system is composed of Endoglucanase, Exo-1, 4-ß-glucanase (exocellobiohydrolase), and ß-glucosidase. Together, cellulases hydrolyze ß-1,4 links in cellulose chains (dos Santos et al. 2022). Endoglucanase catalyses the majority of the cellulose hydrolysis, cleaving the polymer to form short oligosaccharides that are then hydrolyzed by Exo-1,4- ß-cellobiohydrolase and ß-glucosidase, which hydrolyses it into glucose (Prasad et al. 2018).

Cellulases are mainly used in various industries such as; pulp and paper, beer and wine, textile for bio-stoning and bio-finishing, food due to its role in fruit and vegetable juice extraction, detergents to enhance the softness and brightness of clothes, agriculture for controlling plant pathogenesis in addition to improving the nutritional value and digestibility of animal feeds (Behera et al. 2017; Korsa et al. 2023).

Cellulases have drawn a lot of interest lately because of their role in converting cellulosic biomass into useful products. The majority of cellulosic biomass comes from agricultural and industrial wastes (Álvarez et al. 2016; Kumar et al. 2019; Wang et al. 2024). It's important to get rid of these wastes for a variety of sectors. Bioethanol, biomethane, biohydrogen, sugars, and other chemicals could be synthesized by them (Joshi et al. 2019).

A crucial strategy to solve the technical and financial disadvantages of normal enzymatic hydrolysis that have gained attention in recent years is switching from mesophilic to thermophilic enzymes. The depolymerization of complicated lignocellulose polymers is quite interesting when using thermostable cellulase. These cellulolytic enzymes from thermophilic organisms are capable of hydrolyzing chemically pretreated biomass. These enzymes can be released as free enzymes or combined into cellulosomes, complexes of multienzymes that, when put in contact with a cellulose substrate, effectively release sugar (Ajeje et al. 2021). Thermostable cellulases have been produced by numerous thermophilic bacteria such as; *Acidothermus, Caldocellum, Geobacillus, Caldibacillus, Bacillus* (Ghosh et al. 2020; Wang et al. 2024)*,* and *Streptomyces thermodiastaticus* (Suriya et al. 2016)*.*

Microbial enzymes production is affected by the media composition. In addition environmental factors such as temperature, pH, and incubation time play important role in improving the enzyme production by microorganisms. Therefore, environmental and nutritional optimization for microbial growth and enzyme production is essential to obtain high yields of cellulase (Budihal et al. 2016; Korsa et al. 2023). Statistical approaches for optimization using multivariate techniques enable the creation of mathematical models that help in evaluating the relevance and statistical significance of the factor effects under investigation, as well as the evaluation of the factor interactions. As a result, Response Surface Methodology (RSM) is currently commonly utilized for optimization of a wide range of systems due to its high efficiency (Vijayaraghavan et al. 2017; Nisar et al. 2020). Also, improvement of microbial enzyme production can be achieved by introducing random mutations by ultraviolet or gamma irradiation or by treatment with chemical mutagens (Han et al. 2019). Therefore, the aim of the current study involved improvement of cellulase production by a promising thermophilic bacterial strain isolated from Egyptian soil samples, and characterization of the purified cellulase.

## Materials and methods

### Isolation of *Streptomyces* isolate with a high cellulase production

The *Streptomyces* isolate TS4, characterized by high cellulase production, was recovered from an Egyptian soil sample as stated by Gaur & Tiwari ( 2015). The strain was purified by streaking on a CMC agar plate (1.0 g KH_2_PO_4_, 0.5 g MgSO_4_·7H_2_O, 0.5 g NaCl, 0.01 g FeSO_4_·7H_2_O, 0.01 g MnSO_4_·7H_2_O, 0.3 g NH_4_NO_3_, 10.0 g CMC (high viscosity), 20.0 g agar per liter, pH 7.0) followed by 96 h incubation at 49 °C. Then, the purified isolate was maintained over CMC agar slants at 4 °C. A 10% glycerol-containing Brain Heart Infusion (Sigma-Aldrich, USA) stock culture was prepared and kept at  − 80 °C. (Abu Zaid et al. 2021).

### Conditions for bacterial growth and enzyme production

A 5 days-old culture of TS4 isolate was used to prepare a spore suspension in sterile distilled water which was used to inoculate 250 mL Erlenmeyer flasks containing 50 mL CMC liquid medium (1.0 g KH_2_PO_4_, 0.5 g MgSO_4_·7H_2_O, 0.5 g NaCl, 0.01 g FeSO_4_·7H_2_O, 0.01 g MnSO_4_·7H_2_O, 0.3 g NH_4_NO_3_, 10.0 g CMC per liter, pH 7.0), followed by incubation in a rotary shaking incubator (C25, New Brunswick scientific, USA) for 96 h at 49 °C and 200 (x) g. After incubation, 30 ml of culture broth was undergone 20-min centrifugation at 6,000 (x) g. The crude enzyme was obtained from the supernatant to evaluate the cellulase activity by enzyme assay. To calculate the dry cell weight (DCW), the collected cell pellets were dried at 60°C in a hot oven to a consistent weight after being washed twice with sterile saline (Sethi et al. 2013; Yassien et al. 2014).

### Enzyme assay using 3, 5-dinitrosalicylic acid (DNS) reagent

The evaluation of the enzyme activity followed Miller's procedures (1959). In brief, 0.4 mL of crude enzyme solution was mixed with 1.6 mL of 0.5% carboxymethyl cellulose in sodium phosphate buffer (50 mM, pH 7.0), followed by incubation in a shaking water bath (GFL, Germany) at 50 °C for 30 min, then the addition of 3 mL DNS reagent to stop the reaction. The color developed by boiling for 5 min, and stabilized by adding 1 mL 40% potassium sodium tartrate. The mixture's absorbance at 540 nm was measured after cooling using a spectrophotometer (Pharmacia Biotech, England) in comparison to a blank that included all of the ingredients except the crude cellulase. Findings were measured in terms of cellulase activity, where one unit (U) is the cellulase’ quantity that releases 1 µmol of glucose per min. under the circumstances specified for the test. (Budihal et al. 2016; Swathy et al. 2020).

### Molecular identification of TS4 (the selected isolate)

Identification was accomplished using 16S ribosomal RNA gene sequence analysis. A culture was delivered to Sigma Scientific Co. (Cairo, Egypt) for DNA extraction, 16S rRNA gene PCR amplification, and sequencing. The 16S rRNA gene amplification was conducted using the following 2 primers; StrepF: 5-ACGTGTGCAGCCCAAGACA-3 and StrepR: 5-ACAAGCCCTGGAAACGGGGT-3. Afterward, the obtained sequences were submitted to the GenBank database and the DNA similarity was evaluated using the NCBI BLAST® (http://blast.ncbi.nlm.nih.gov/Blast.cgi). Sequence alignment had been performed. The phylogenetic analysis for the TS4 isolate's 16S rRNA gene was generated by the Neighbor-joining approach using MEGA 11.0 software to study the genetic relatedness of the chosen *Streptomyces* isolate (TS4) in the current study to other *Streptomyces* strains in the Genebank database. (Kumar et al. 2018). Also, the 16S ribosomal RNA sequence was added to the World Data Centre for Microorganisms (WDCM) Culture Collection Ain Shams University (CCASU).

### Effect of different environmental and nutritional factors on cellulase production by the selected isolate

Several environmental factors such as incubation temperature (40, 45, 49, and 55 °C), agitation speed (100–250 (x) g with an interval of 50 (x) g), initial pH of fermentation medium (5–9 with an increment of 1), the incubation period (3–7 days) and inoculum size (0.5–2% v/v with an increment of 0.5%) were assessed for their effect on cellulase production and microbial growth as described before. In addition, effect of some media components such as nitrogen sources (ammonium nitrate, yeast extract, tryptone, ammonium chloride, peptone and soybean—0.03% w/v) (Budihal et al. 2016), CMC concentrations (0.5–3% w/v) (Islam and Roy 2018) and mineral salts (ZnSO_4_, CaCl_2_, MnSO_4_, NaCl, MgSO_4_ and FeSO_4_—0.1% w/v) (de Cassia Pereira et al*.*^2017^; Ahmad et al. 2020) were also investigated. At first, the OFAT technique was followed to find the crucial process variables affecting cellulase production. Cellulase activity and DCW were determined as mentioned before.

### Improvement of cellulase production using response surface design

To maximize enzyme production, the ideal combination and interplay of crucial process parameters were determined using the Central Composite design (CCD). Initial pH, concentrations of CMC, tryptone, and sodium chloride in the fermentation medium were determined as essential process parameters from the preliminary studies. The incubation time and inoculum volume were kept constant since they did not affect cellulase production. Design Expert® v. 7.0 (Design Expert® Software, Stat-Ease Inc., Statistics Made Easy, Minneapolis, MN, USA) was used to create a CCD design matrix with 30 experiments and six replicate center points. The dependent variable or response (Y) was the average maximum cellulase activity, while the independent ones were the essential process parameters. The codes and values of the three levels for the four studied variables are shown in Table 1. The predicted response was calculated using a second-order polynomial equation created by the software that accepts all interaction terms. The 3D plots were created to describe the correlations between the four experimental variables and cellulase activity.

**Table 1:** The independent parameters’ levels studied by CCD

Test parameter	Parameter code	Parameter levels		
		− 1	0	1
Initial pH	A	6	6.5	7
CMC Conc. (%)	B	1	1.5	2
Tryptone Conc. (%)	C	0.03	0.065	0.1
NaCl Conc. (%)	D	0.075	0.1	0.13

### Validation of the applied model data

To predict the maximum response values, Design-Expert's numerical optimization tool was used. According to the obtained results of conducted lab experiments, the validity of the model can be measured based on the degree of similarity between observed lab data and predicted model data. The values achieved under optimized conditions were compared to those obtained under non-optimized conditions.

### Improvement of cellulase production of the selected isolate by mutation with gamma irradiation

According to Khaliq et al*.* (2009) using some alterations, the TS4 isolate was subjected to gamma radiation to undergo mutation. A 1 × 10^8^ CFU/mL bacterial suspension was prepared and exposed to gamma radiation at various doses [1, 2, 3, 4, and 5 Kilo Gray (KGy)]. The used source of gamma radiation was the Indian Gamma cell’s 60Co producing doses at a rate of 1.43 KGy/h. The operation was conducted at the Atomic Energy Authority's National Center for Radiation Research and Technology in Nasr City, Cairo, Egypt. Following irradiation, a random selection of recovered colonies was purified and stored on CMC slants. Comparing the selected colonies' cellulase activity to that of the wild strain was done using DNS assay.

### Partial purification of cellulase produced by TS4 isolate

To achieve 80% saturation, the addition of (NH4)2SO4 to the supernatant containing the crude cellulase was performed (Islam and Roy 2018; Lübeck 2018; Swathy et al. 2020). The resulting mixture was maintained at 4 °C overnight with steady stirring. Then the pellets were collected by centrifuging this mixture at 12,000 (x) g for 20 min at 4 °C and reconstituted in phosphate buffer (100 mM, pH 7.0). The suspension was dialyzed using a 12–14 kDa cut-off membrane (Spectra/Por dialysis membrane, which was obtained from Spectrum Laboratories Inc., Rancho Dominguez, Canada) against 1L of the same buffer under stirring at 4 °C for 48 h with one change after 24 h. Using the Lowry method, which utilized bovine serum albumin (BSA) as a control, the protein content was measured (Lowry et al. 1951).

### Sodium dodecyl sulphate polyacrylamide gel electrophoresis (SDS-PAGE) and zymogram analysis

This was carried out utilizing 12% resolving gel as described by Lübeck (2018). Zymogram analysis of cellulase required the addition of 0.1% (w/v) CMC to the resolving gel. The gel was split into two portions after electrophoresis; one was utilized for Coomassie brilliant blue R-250 staining and the other for zymogram analysis. For zymogram analysis, the second part of the gel was rinsed for an hour with 1% (v/v) Triton X–100 in sodium citrate buffer (50 mM, pH 5.5). The buffer solution was removed and replaced with another fresh aliquot of the same buffer, followed by incubation for four hours at 50 °C. It was then dipped into Congo red solution (0.1% w/v) for 30 min before being rinsed with 1 M NaCl for 1 h or until the clear zones around the enzyme were detected. For molecular weight estimation, a Prestained Protein Ladder (245 kDa) (Geneaid Biotech Ltd, Taiwan) was used.

### Enzyme characterization

#### Impact of pH and temperature on enzyme activity

The impact of pH on enzyme activity was investigated by incubating the enzyme assay reaction mixture at different pH values (4.0–10.0). This was done by forming 0.5% CMC in 50 mM of different buffers; sodium citrate for pH 4.0 and 5.0, sodium phosphate for pH 6.0, 7.0, and 8.0, and glycine NaOH for pH 9.0 and 10.0. Then the activity was quantified as relative activity compared to that at pH 7.0 (Swathy et al. 2020). To find the optimum temperature for maximal cellulase activity, the reaction assay incubation temperature was varied from 40 to 90 °C. The cellulase activity was calculated as a percentage of its activity at 50 °C (Gaur and Tiwari 2015).

### Effect of temperature on enzyme stability

Different temperatures were applied to the purified cellulase preparations (50, 60, 70, 80, and 90 °C) for 0.5 h, 1 h, and 12 h, then immediately cooled. Residual activities were calculated as percentages compared to those of untreated enzyme preparations (Akintola et al. 2017).

### Statistical analysis

Each experiment was carried out three times, and the reported findings are the means ± the standard deviation. To analyze the data and generate the 3D and the model diagnostic plots, Design Expert® v. 7.0 was employed. ANOVA, which uses reported P-values to show the importance of each parameter, was used to statistically validate the experimental data.

## Results

### Identification of TS4 isolate

**Fig. 1:**
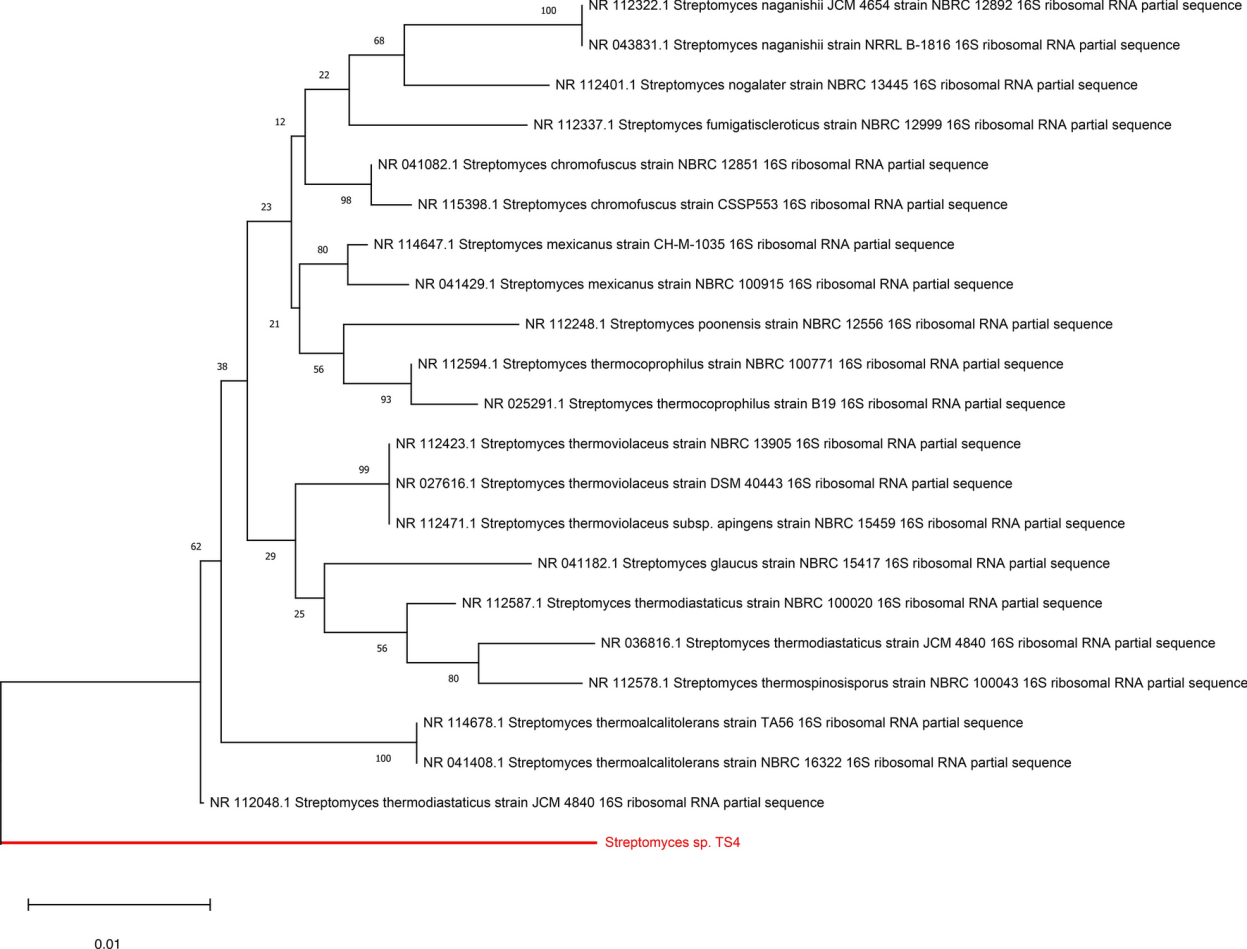
The phylogenetic tree of *Streptomyces* TS4 (labelled by the red colour) and related species based on partial 16S rRNA gene sequences. The tree was created using the MEGA 11.0 software based on the Neighbor-joining method. The bar length represents 0.01 substitutions per nucleotide site

The 16S rRNA gene sequence of TS4 was submitted to the GenBank under accession number of PQ097747. Also, TS4 was added to the World Data Centre for Microorganisms (WDCM) Culture Collection Ain Shams University (CCASU) (strain number, CCASU-2023–61). TS4 isolate was identified as *Streptomyces thermodiastaticus* as it had 96.00% similarity with *S. thermodiastaticus* strain JCM 4840(accession number = NR112048.1). Figure 1 shows the phylogenetic tree of the TS4 isolate.

### Effect of different factors on cellulase productivity by the selected isolate

**Fig. 2:**
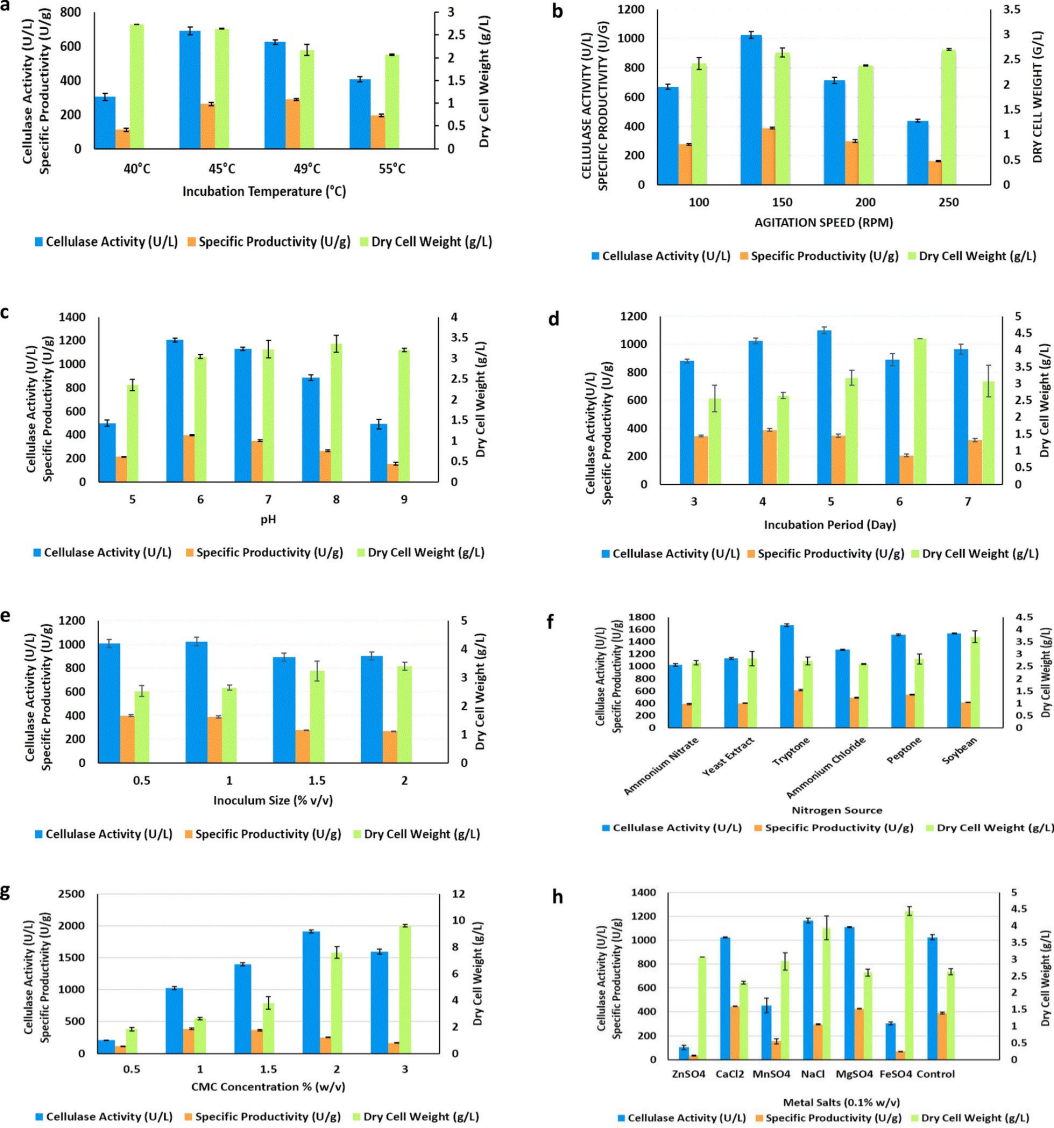
Effect of **a** incubation temperature, **b** agitation speed, **c** initial pH of fermentation medium, **d** incubation period, **e** inoculum size, **f** different nitrogen sources, **g** CMC concentration, and **h** different metal salts on growth and cellulase production by *S. thermodiastaticus*. Results are the mean of 3 observations and error bars show the standard deviation

Regarding the effect of the initial pH of the medium, incubation temperature, and agitation speed, the maximum level of cellulase productivity was obtained at pH 6.0, 45 °C, and 150 (x) g, respectively. In the case of incubation time, it was gradually increased reaching the maximum level after 4 days, after which no significant increase was observed. Accordingly, 4 days incubation period was selected for further studies. Also, the highest level of cellulase productivity was obtained by using a 1% (v/v) inoculum size. Regarding the medium components, the highest level of enzyme productivity by *S. thermodiastaticus* was obtained in the presence of tryptone (as nitrogen source), CMC, and NaCl at concentrations of 0.03%, 2%, and 0.1% (w/v), respectively (Fig. 2).

### Optimization of cellulase production using response surface methodology (RSM) experimental design

Table 2 displays the layout, the experimental findings, and the anticipated values by the second-order polynomial equation that was fitted by the Design-Expert program. The following Equation was fitted for cellulase activity.

**Table 2:** The central composite design for the 4 different factors with observed and predicted responses

Run order	pH	CMC conc. (%)	Tryptone conc. (%)	NaCl conc. (%)	Cellulase activity (U/L)	
					Predicted response	Observed response
1	6.5	1.5	0.07	0.1	1226.31	1143
2	6	2	0.03	0.13	2007.3	2034
3	6	2	0.1	0.13	1743.51	1787
4	6	2	0.03	0.08	1967.88	1976
5	7	1	0.03	0.13	733.76	766
6	7.5	1.5	0.07	0.1	866.36	750
7	7	1	0.1	0.13	726.71	714
8	6.5	0.5	0.07	0.1	407.53	327
9	6.5	1.5	0.07	0.1	1226.31	1119
10	5.5	1.5	0.07	0.1	1306.2	1295
11	6.5	1.5	0.14	0.1	1090.9	1239
12	7	1	0.03	0.08	694.34	860
13	6	1	0.1	0.13	816.26	825
14	6	1	0.03	0.13	823.3	818
15	7	2	0.03	0.08	1617.59	1694
16	6.5	2.5	0.07	0.1	2258.03	2211
17	6.5	1.5	0.07	0.15	1265.73	1214
18	6.5	1.5	0.07	0.1	1226.31	1345
19	7	2	0.1	0.08	1353.8	1333
20	6	1	0.03	0.08	783.88	854
21	6	2	0.1	0.08	1704.09	1684
22	7	2	0.1	0.13	1393.21	1383
23	6.5	1.5	− 0.01	0.1	1361.73	1273
24	6.5	1.5	0.07	0.1	1226.31	1222
25	6.5	1.5	0.07	0.05	1186.9	1081
26	6.5	1.5	0.07	0.1	1226.31	1093
27	6.5	1.5	0.07	0.1	1226.31	1279
28	7	2	0.03	0.13	1657.01	1775
29	7	1	0.1	0.08	687.3	699
30	6	1	0.1	0.08	776.84	795

Final Equation applying Actual Factors:

Cellulase activity =  − 7261.88765 + 1991.61458 × pH + 2539.12946 × CMC conc. + 3567.26190 × tryptone conc. + 788.33333 × NaCl conc.—260.75000 × pH x CMC conc.—3667.85714 × CMC conc. x tryptone conc.—140.03125 × pH^2^ + 106.46875 × CMC conc.^2^

Table 3 reveals the ANOVA results that check the models' accuracy and discusses the relevance of the variables and their impact on cellulase production. For cellulase production, the F value of the model was 85.31 (*P*-value < 0.0001) indicating that it is valid. Moreover, it was observed that the model terms A, B, C, AB, and BC (*P*-value < 0.05) were significant. A low value of the coefficient of variation (CV) of 7.52% indicates the good reliability of the experimental values. The coefficient of determination, R^2^, was 0.9701, suggesting that the model could account for 97.01% of the variation in the outcome. A PredR^2^ of 0.9349 was determined to be reasonably consistent with the AdjR^2^ which was found to be 0.9588. The lack of fit is not significant in comparison to the pure error, according to the "Lack of Fit F-value" of 0.81. The model fits if there is no notable lack of fit. Finally, the signal-to-noise ratio, known as adequate precision, was 36.834 and revealed a sufficient signal. Consequently, the model can be utilized to explore the design space.

**Table 3:** ANOVA for response surface reduced quadratic model for cellulase production

Source	Sum of squares	Df	Mean square	F value	*p*-Value	
Model	5.742E + 006	8	7.177E + 005	85.31	< 0.0001	Significant
A-pH	2.902E + 005	1	2.902E + 005	34.49	< 0.0001	
B-CMC conc	5.137E + 006	1	5.137E + 006	610.53	< 0.0001	
C-tryptone conc	1.100E + 005	1	1.100E + 005	13.08	0.0016	
D-NaCl conc	9322.04	1	9322.04	1.11	0.3045	
AB	67,990.56	1	67,990.56	8.08	0.0097	
BC	65,920.56	1	65,920.56	7.84	0.0107	
A^2^	34,860.00	1	34,860.00	4.14	0.0546	
B^2^	20,152.17	1	20,152.17	2.40	0.1366	
Residual	1.767E + 005	21	8413.20			
Lack of fit	1.277E + 005	16	7979.27	0.81	0.6589	Not significant
Pure error	49,008.83	5	9801.77			
Cor total	5.918E + 006	29				

**Fig. 3:**
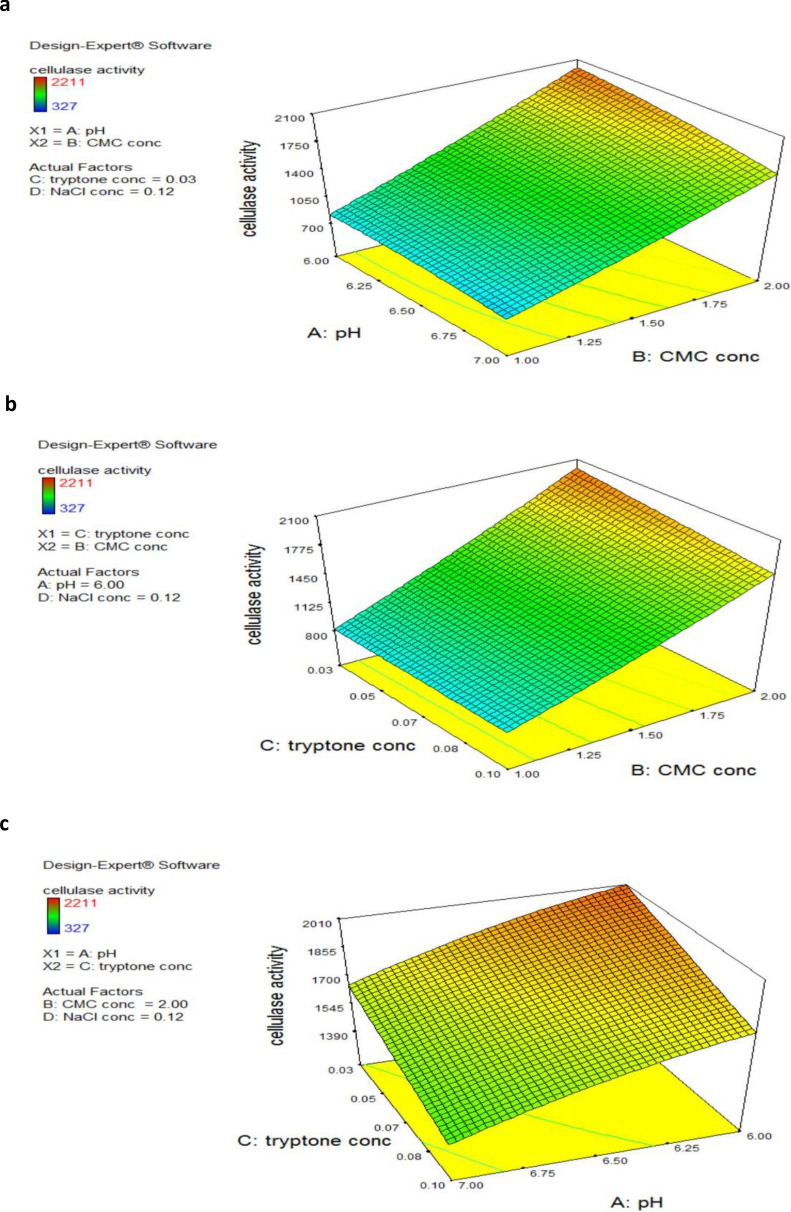
The 3D response surfaces showing the four crucial variables’ impact on cellulase production of *S. thermodiastaticus***a** pH, CMC concentration, and tryptone concentration are fixed at optimum levels **b** CMC concentration, tryptone concentration, and pH is fixed at optimum levels **c** tryptone concentration, pH and CMC concentration is fixed at optimum levels

The optimal conditions for cellulase production were determined to be a pH of 6.0, CMC conc. of 2%, tryptone conc. of 0.03%, and NaCl conc. of 0.12% based on the three-dimensional surface plots (Fig. 3) and the numerical optimization tool in the Design-Expert program.

**Fig. 4:**
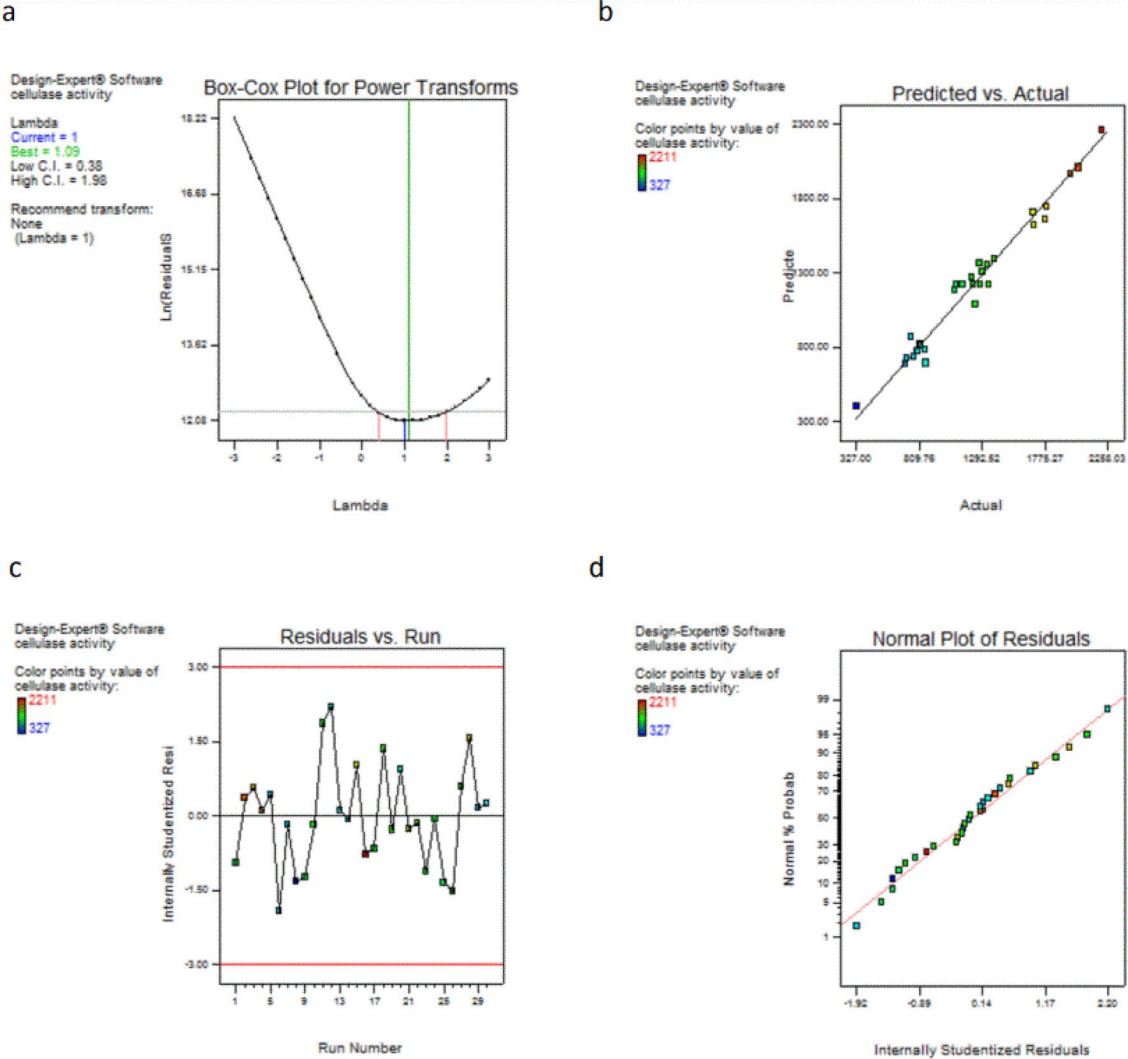
Model diagnostic plots **a** Box-Cox plot, **b** Predicted vs. actual plot, **c** Residuals versus Run number plot, and **d** The normal probability plot of residuals

The following graphical summaries were created to validate our models:

A Box-Cox plot is an effective tool for determining the most appropriate power transformation. It demonstrated that the current lambda (= 1) was sufficient and that no further change was required (Fig. 4a).

The predicted vs. actual plot revealed that the predicted and experimental results were in good correlation (Fig. 4b).

The residuals versus Run number plot (Fig. 4c) demonstrated that the model matches the data as the points were scattered around zero.

The residuals' normal plot (Fig. 4d) revealed that the residuals were normally distributed as the points construct a linear graph.

### Experimental confirmation test

Cellulase activity of 2023 U/L, which was produced utilizing the indicated ideal levels of the four variables, was extremely similar to that anticipated by the utilized model (2005.16 U/L). These results demonstrate that the model is reliable, accurate, and useful for forecasting *S. thermodiastaticus*'s cellulase production. As a result, the cellulase activity was 3.24 times higher under the optimized circumstances than it was under the basal ones (625 U/L).

### Strain improvement

Cellulase productivity of *S. thermodiastaticus* was improved using gamma irradiation. Regarding the results, exposure to 4 KGy gamma radiations allowed the selection of 3 mutants (M1, M2, and M3) with higher cellulase productivity (984.63, 839.62, and 942.81 U/L, under basal conditions, respectively) in comparison to that of the wild strain (984 U/L). When M1 (highest enzyme producer mutant) was studied for enzyme productivity under the optimized condition, the enzyme production reached 3187.23 U/L which is approximately 1.5 times as much as that of the wild-type strain (2023U/L).

### Cellulase partial purification from the culture supernatants

**Fig. 5:**
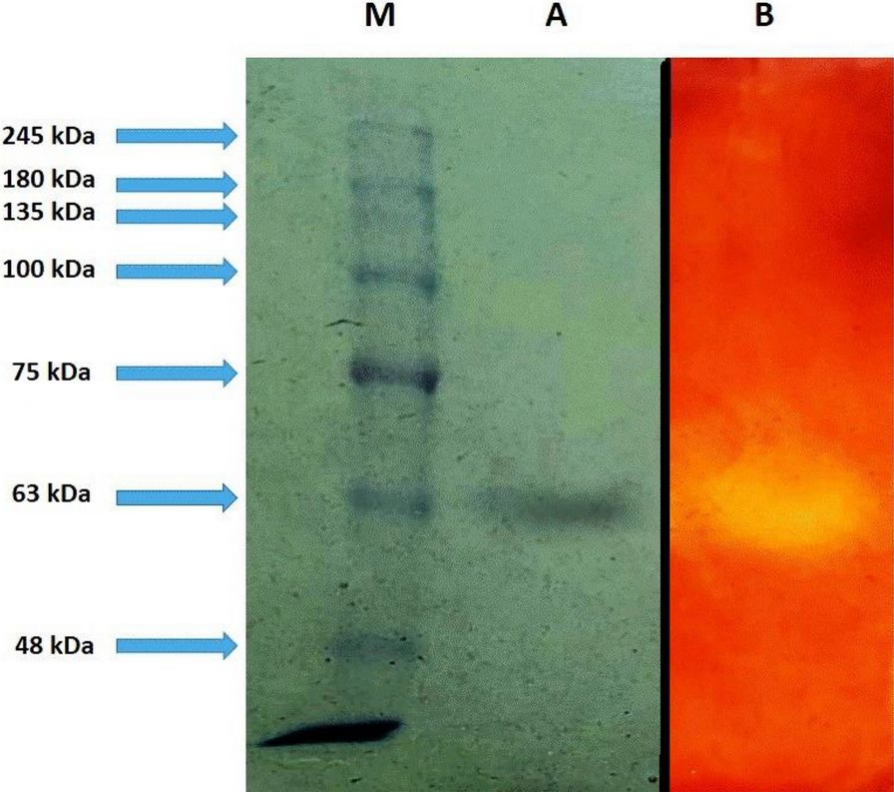
SDS-PAGE showing purified cellulase from *S. thermodiastaticus*. Lane M: Molecular mass marker (Prestained Protein Ladder (245 kDa), Geneaid), Lane A: Purified cellulase (stained by Coomassie brilliant blue), and Lane B: Purified cellulase’ zymogram (stained by Congo red)

The partially purified enzyme was obtained after ammonium sulfate precipitation and dialysis. After each step of partial purification, determination of protein content, analysis by SDS-PAGE, and quantitative determination of enzyme activity were carried out (Table 4). According to the obtained SDS-PAGE and zymogram results, a single band at molecular weight 63 kDa was obtained after ammonium sulfate precipitation and dialysis (Fig. 5). In addition, an increase in the enzyme activity by 1.74 times as compared to the crude extract was achieved.

**Table 4:** Cellulase partial purification from the selected *S. thermodiastaticus* isolate

Purification steps	Volume of fraction (ml)	Activity (U)	Protein (mg)	Specific activity (U/mg protein)	Purification (fold)
Crude enzyme	825	697.125	288.6	2.416	1
Ammonium sulfate precipitation and dialysis	22	74.104	17.6	4.210	1.74

### Enzyme characterization

**Fig. 6:**
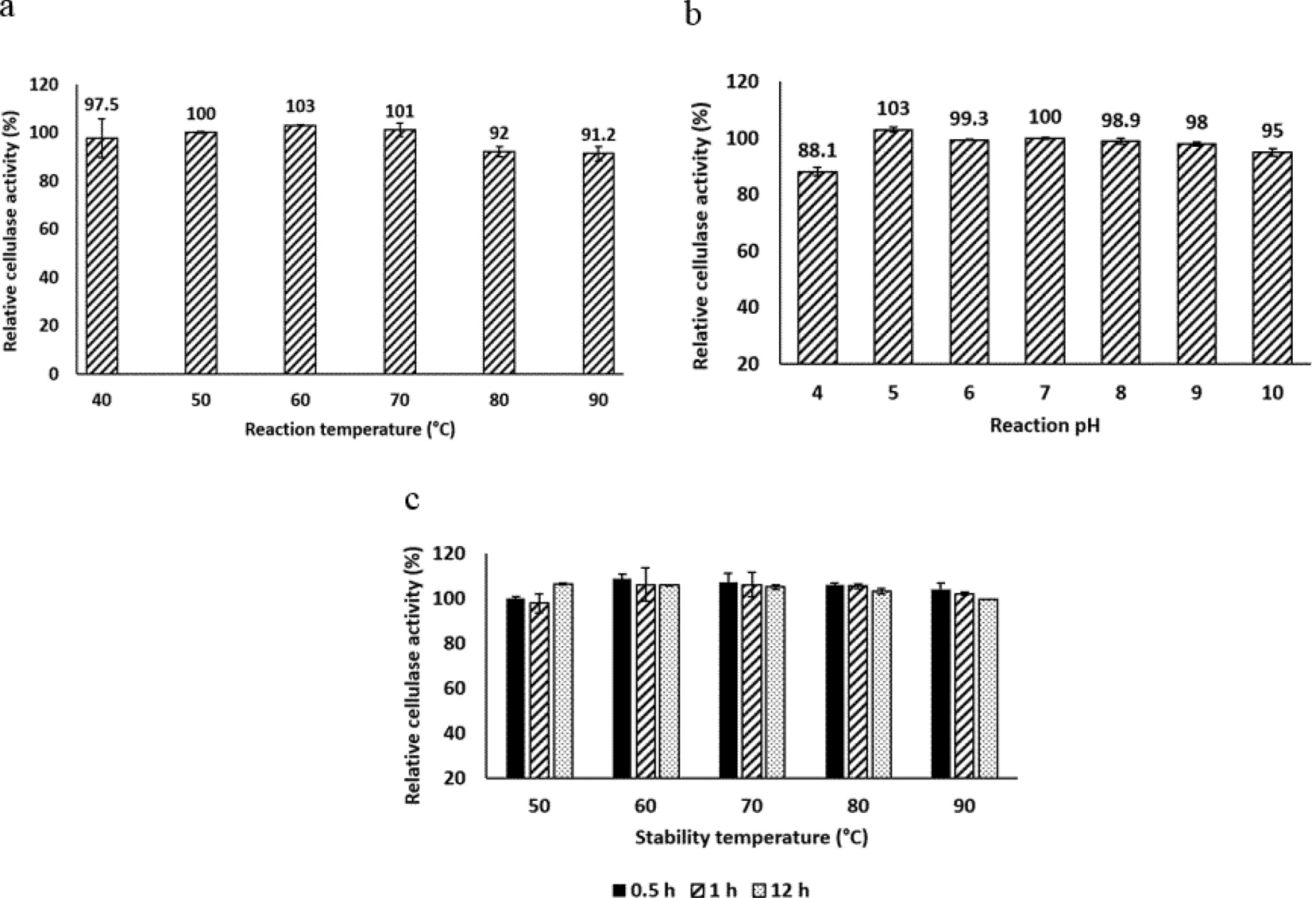
Relative activities at **a** different temperatures, **b** pH values, and **c** thermal stability of cellulase produced by *S. thermodiastaticus*. The mean of 3 separate experiments was used to get the results while the error bars show the standard deviation

The maximum cellulase activity was achieved by using a 60°C incubation temperature. At a higher incubation temperature (70–90 °C), cellulase could preserve more than 90% of its activity (Fig. 6a). Moreover, pH 5 was found optimum for the enzyme activity and it could maintain ˃95% of its activity over the pH range 6–10 (Fig. 6b). Regarding thermostability, thermal treatment of the enzyme at temperatures ranging from 50 to 90 °C for 0.5, 1, and 12h showed no significant change in cellulase activity (Fig. 6c).

## Discussion

Because of cellulase’s numerous uses in various industrial sectors, it is considered one of the most important industrial enzymes. The value of the global cellulase market was USD 1820.5 million in 2020, and by 2027, it is anticipated to reach USD 2345.2 million, with a Compound Annual Growth Rate of 6.5% between 2021 and 2026 (LP Information, Inc., 2021). Thermostable cellulases have specific advantages over their mesophilic alternatives, such as high catalytic activity, thermostability, tolerance to high pressure and chemical denaturants, and reduction of product contamination risks (Akram et al. 2018; Ajeje et al. 2021). In addition, at rising temperatures, the rapid diffusion rate of nutrients and low viscosity enhance microbial growth and production while reducing bio-contamination hazards (Cuecas et al. 2016; Ebaid et al. 2019). Furthermore, a major shift in biofuel production is the employment of thermophilic microorganisms and their products, because of the efficient exploitation of lignocellulosic materials (Dai et al. 2014). Fortunately, our test isolate was identified as *Streptomyces thermodiastaticus*, a thermophilic strain, by the use of 16S ribosomal RNA gene sequencing which is the most common technique for bacterial detection (Johnson et al. 2019).

Optimization of the nutritional and environmental parameters of fermentation processes is required to increase microbial biomass and subsequent cellulase production (Latha et al. 2017; Arora et al. 2020; Sutaoney et al. 2024). In the present study, the classical method (OFAT) (which involves modifying one independent variable while keeping all other variables constant) was used to select the factors with a high impact on cellulase production, followed by the statistical one (RSM) to detect the correlation between the selected factors. Incubation temperature is critical for the optimum production of enzymes as temperature fluctuations can alter the structure and characteristics of bacterial proteins (Juturu and Wu 2014; Nisar et al. 2020). Extracellular enzyme secretion was also discovered to be influenced by temperature, probably due to changes in the physical properties of the cell membrane (Sethi et al. 2013). For the production of cellulase by *S. thermodiastaticus* (tested in the present study), The optimum temperature was 45°C which is similar to that of *Streptomyces* DSK59 (Budihal et al. 2016). Also, 40°C was recorded for different *Streptomyces* sp. (Fatokun et al. 2016; Sinjaroonsak et al. 2020) while 50°C was found to be suitable for cellulase production by *S. viridobrunneus* (Da Vinha et al. 2011). Differences in the optimum temperature for enzyme synthesis could be due to strain differences and their capacity to respond to temperature changes (Fatokun et al. 2016; Wang et al. 2024).

A significant aspect that may have an impact on the productivity of microbial enzymes is agitation. During the fermentation process agitation is responsible for mixing, which allows heat, nutrients, and oxygen to be fully mixed and efficiently transferred into the fermentation broth. Mycelia clustering is also prevented by agitation, allowing for better oxygen uptake (Balan et al. 2013; Xia et al. 2014). The optimum agitation speed for maximum enzymatic production by *S. thermodiastaticus* was 150 (x) g which is consistent with that stated by Ahmad et al*.* (2020) and Swathy et al*.* (2020). On the other hand, a higher agitation speed (300 (x) g) was reported as the optimal speed for cellulase production by other *Streptomyces* sp. (Jang and Chang 2005).

The initial pH of the culture medium has an impact on enzyme production and transfer through the cell membrane (Sinjaroonsak et al. 2020). The initial pH of 6.0 was found to be optimum for cellulase production by the tested isolate which is in agreement with that reported previously for other *Streptomyces* sp. (Budihal et al. 2016; Fatokun et al. 2016; Sinjaroonsak et al. 2020). Other researchers documented pH 5.0 (Rathnan 2011), pH 6.5 (Josh and Mahawar 2022) and pH 7.0 (Islam and Roy 2018; Ahmad et al. 2020) as optimal for cellulase production.

During the incubation period of fermentation, it was observed that the maximum enzyme production by the tested isolate was achieved after 96h. A longer incubation period was related to a reduction in enzyme production by microorganisms. This could be owing to the medium's depletion of macro-and micronutrients over time that stressed the bacterial physiology leading to inactivating the enzyme-secreting machinery. Another explanation could be the medium's high viscosity, which restricts oxygen availability to the microbes (Mai 2018). The same optimum incubation time of 96 h was reported previously (Budihal et al. 2016). A longer incubation period, more than 5 days, was mentioned by Sadhu et al. (2014) and Sinjaroonsak et al. (2020), while only 60 h was recorded for *Streptomyces albidoflavus* (Fatokun et al. 2016).

For each strain, it is crucial to study the inoculum size as it may have an impact on the productivity of the enzymes. Since, low inoculum size results in a small proportion of microbial cells in the production medium, it takes a long time for the microbial cells to achieve their maximum growth number and create the desired output. Up to a certain degree, a large inoculum size normally boosts bacterial growth and its related activities; but, beyond that point, the rapid microbial growth may cause a drop in bacterial activity as it causes a shortage of total dissolved oxygen and nutrition supply to the bacteria (Hasan et al. 2017). In this investigation, the maximum cellulase productivity was achieved by using a 1% (v/v) inoculum size.

The nitrogen supply and its level in the medium have an impact on the synthesis of cellulases (Sharma et al. 2021). Optimum cellulase production occurred when tryptone was used as a nitrogen source at a concentration of 0.03% w/v. This conclusion was in agreement with previous findings by Chellapandi and Jani (2008). Different nitrogen sources were reported for optimal cellulase production by other microorganisms such as; ammonium nitrate for *Bacillus* sp. (Sadhu et al. 2014), peptone for *Paenibacillus* sp. (Islam and Roy 2018), yeast extract for *Streptomyces thermocoprophilus* (Sinjaroonsak et al. 2020), and ammonium chloride for *Aneurinibacillus aneurinilyticus* (Ahmad et al. 2020). Regarding microbial growth, no significant increase was observed with the tested nitrogen sources as compared to the original one except for soybean, which is recognized by the presence of insoluble components. According to previous reports, CMC is considered the most suitable carbon source for microbial production of cellulase enzyme, in addition to its inducer effect (Sinjaroonsak et al. 2020; Mokale Kognou et al. 2022), therefore it was used in the present study. The increase in CMC concentration from 0.5 to 2% (w/v) was associated with an increase in cellulase production. This finding is consistent with previous research indicating that CMC induces cellulase synthesis and serves as a preferred substrate for cellulase production (Islam and Roy 2018). However, higher CMC concentration (> 2%) caused a reduction in the enzymatic activity, which may be related to the inhibitory effect of accumulating cellobiose and cellodextrin (Swathy et al. 2020).

Regarding the effect of metal salts on bacterial growth and cellulase production, optimum cellulase activity was recorded in the presence of sodium chloride which was consistent with that stated by Budihal et al. (2016). Although zinc sulfate, manganese sulfate, and ferrous sulfate had a highly positive impact on bacterial growth, they dramatically decreased cellulase activity to 10%, 44%, and 30%, respectively, of that of the control. Their inhibitory action could be explained by metals binding to cellulase, causing conformational changes or the substitution of native cofactors, or by metals binding to cellulose, preventing cellulase access (Singh et al. 2015). The inhibitory effect of these metal ions was observed previously by other investigators (Shanmughapriya et al. 2010; Tao et al. 2010; Gaur and Tiwari 2015).

Statistical approaches for optimization using multivariate techniques have been recommended, as they are faster, more cost-effective, and allow for simultaneous optimization of several variables. Furthermore, these methodologies permit the assessment of the signif +  + 3 + 0.3icance of the factor effects under investigation, as well as the evaluation of the factor interactions. As a result, RSM is currently commonly utilized for the optimization of a wide range of systems due to its high efficiency (Nisar et al. 2020).

The CCD, one of the most widely used and effective designs, is perfect for subsequent analysis with a manageable number of run (El-Sayed et al. 2020). To investigate the impact of the four selected factors (initial pH, CMC concentration, tryptone, and sodium chloride) on cellulase production, a series of 30 experiments were undertaken with various combinations of independent variables including 6 replicates at the center points. With an F-value of 85.31 and a (P _model_ > F) = 0.0001), the inferred model was extremely relevant. There is only a 0.01% chance that a "Model F-Value" this large will be caused due to noise. The observational results were exceptionally precise and reliable, as seen by the low CV value of 7.52 percent. (Nisar et al. 2020). In comparison to the pure error, the "lack-of-fit F-value" of 0.81 shows that it is not significant (Thakkar and Saraf 2014). R2 is 0.9701, indicating that the model also provided a very good fit between the actual and expected responses (Luo and Chen 2010). Also, it was close to the predR^2^ which equals 0.9349, indicating that the experimental data for the independent variables was well fitted with the model's predicted values. The adj R^2^, which measures if extra input variables contribute to the model, equals 0.9588, was very close to both R^2^ and predicted R^2^ indicating that the model is better fitted, reliable, and predictable (Vijayaraghavan et al. 2017). Similar results were reported by (Thakkar and Saraf 2014; Budihal et al. 2016; Nisar et al. 2020). The signal-to-noise ratio was calculated using the term "adequate precision." To be considered sufficiently accurate, a model must have a value of > 4.0. The model used for the optimization of cellulase production by *S. thermodiastaticus* has an adequate precision of 36.834, which is much higher than the required level for the model to be deemed suitable and effective to explore the design space. (Thakkar and Saraf 2014).

The P-value was utilized to assess each component's relevance (Pal et al. 2009; Thakkar and Saraf 2014). Significant model terms have P-values lower than 0.0500 (Nisar et al. 2020). These results determined that the production of cellulase by *S. thermodiastaticus* was significantly influenced by initial pH (A), CMC concentration (B), tryptone concentration (C), and the interactive effects of AB and BC. The 3D and their corresponding contour plots provide not only rich information on the relationships between different variables but also a straightforward forecast of the optimized laboratory circumstances (Ghribi et al. 2012). These plots and a numerical optimization tool were used to identify the indicated ideal conditions for the highest cellulase production, which were then tested practically. The results confirmed the validity and reproducibility of the optimal conditions for *S. thermodiastaticus* using the designed model. Finally, these findings demonstrated that experimental design and RSM are effective methods for analyzing the environmental circumstances for cellulase production. Using RSM,* S. thermodiastaticus* production of cellulase was increased by about 3.24 fold as compared to that obtained under basal conditions (625 U/L).

Random mutagenesis had been indicated as an effective way for strain enhancement to increase the metabolic activity of microbes (Zia et al. 2012; Korsa et al. 2023). Gamma rays were used to carry out the mutagenesis in this work (physical mutagen). Gamma rays are the most potent and intense type of ionizing radiation. They cause a mutation by breaking single and double-stranded DNA, resulting in structural alterations or oxidation (Elkenawy et al. 2017). The induced mutation using gamma radiation had been claimed to be an efficient way to improve enzyme production by different microbial strains (Iftikhar et al. 2010; Zia et al. 2012; Lv et al. 2013). Finally, improvement of the cellulase productivity of the chosen strain, *S. thermodiastaticus*, by nutritional and environmental optimization as well as genetic manipulation enhanced the cellulase productivity by 5.1 times in comparison to that produced by the wild-type strain under the initial conditions.

Ammonium sulfate precipitation followed by dialysis was used to partially purify cellulase from the selected isolate, *S. thermodiastaticus*. A rise in the purification fold to 1.74 with a specific activity of 4.21 U/mg was accomplished along with the processes for enzyme purification. Approximately similar specific activity (3.77 and 5.28 U/mg) was obtained after enzyme purification from the culture broth of *Consortium* XM70 and *Cellulomonas uda* (Zhao et al. 2015; Swathy et al. 2020), respectively, although both studies complete the purification steps by Sephadex and/ or ion exchange resin. The recovered cellulase enzyme has a molecular weight, of 63 kDa, close to the cellulase enzyme produced by *Trichoderma viride* (58 kDa), *Paenibacillus* sp. (67 kDa), and *Cellulomonas uda* (64 kDa) (Iqbal et al. 2011; Islam and Roy 2018; Swathy et al. 2020), respectively. However, cellulase enzyme with a smaller molecular weight was produced by *Streptomyces longispororuber* (42 kDa) (Yassien et al. 2014). In addition, cellulase enzyme with a larger molecular weight was produced by *Streptomyces viridobrunneus* (119 kDa) and *Bacillus vallismortis* RG-07 (80 kDa) (Da Vinha et al. 2011; Gaur and Tiwari 2015), respectively.

Further studies were carried out to achieve the optimum conditions of the enzymatic activity for the recovered enzyme. One of the most critical parameters in determining cellulase activity is pH. This can be attributed to the cellulase protein's various alkaline and acidic groups, such as amino and carboxyl, which make it very sensitive to severe pH settings. In an acidic or alkaline environment, changes in protein structure, enzyme–substrate dissociation state, and the ionization state in the active center might cause the enzyme to denature (Swathy et al. 2020). The optimum pH of cellulase activity by *S. thermodiastaticus* is 5.0. The same result (pH 5.0) was reported by other investigators (Da Vinha et al. 2011; Singh et al. 2015). In addition, it could retain ≥ 95% and 88.1% over the pH range of 6.0–10.0 and pH 4.0, respectively. On the other hand, a more acidic pH (4.0) was reported as optimum for the cellulase activity by *S. malaysiensis* (Nascimento et al. 2009). Higher optimum pH was reported for the following microorganisms; *Bacillus subtilis* YJ1(pH 6.0–6.5) (Yin et al. 2010), *Streptomyces longispororuber* (pH 6.0–6.5) (Yassien et al. 2014), *Bacillus vallismortis* (pH 7.0) (Gaur and Tiwari 2015) and *Trichoderma viride* (pH 8.0) (Iqbal et al. 2011). Regarding the impact of temperature on cellulase activity, maximal activity occurred at temperatures ranging between 40 and 70°C. At higher temperatures (80–90°C), the enzyme could retain more than 90% of its activity. Maximum cellulase activity was recorded at 50°C by Tao et al*.* (2010) and Swathy et al*.* (2020). While 65–70°C was reported as optimum for the cellulase activity produced by thermophilic *Bacillus vallismortis* RG-07 and thermophilic *Consortium* XM70 (Gaur and Tiwari 2015; Zhao et al. 2015), respectively. By studying the thermo-stability of cellulase enzyme produced by *S. thermodiastaticus*, the results showed no significant change in the enzymatic activity over a wide temperature range (50–90°C) up to exposure time of 12 h. Give an indication for good themal stability of the produced enzyme. Many studies on the thermal stability of thermostable cellulase have been conducted. After incubation for 1h, the thermostable cellulase obtained from *Bacillus vallismortis* RG-07 was completely stable at a wide temperature range of 50–85°C and could retain 95% of its activity at 90°C (Gaur and Tiwari 2015). Furthermore, Zhao et al*.* (2015) reported that the thermostable cellulase produced by *Consortium* XM70 could retain approximately 90% and 70% of its maximal activity after incubating at 70 and 80 °C for 1h, while almost total activity lost at 90 °C after 40 min. These superior properties of outstanding thermal stability and broad pH-optimum make the cellulase enzyme obtained from *Streptomyces thermodiastaticus* an excellent candidate for industrial processes especially for the hydrolysis of cellulosic biomass and biofuel production because of the potential advantages of the thermostable cellulases in this process as discussed previously. Accordingly, this study calls for additional research to produce this beneficial enzyme on a wide scale.

## Data Availability

The published article contains all of the data generated or analyzed during this investigation. New Sequences identified in this investigation have been deposited in NCBI GenBank with the accession number PQ097747 (http://www.ncbi.nlm.nih.gov). Also, TS4 was added to the World Data Centre for Microorganisms (WDCM) Culture Collection Ain Shams University (CCASU) (strain number, CCASU-2023–61).

## Abbreviations

CMC: Carboxymethyl cellulose
DCW: Dry cell weight
DNS: 3, 5-Dinitrosalicylic acid
OFAT: One-factor-at-a-time
CCD: Central composite design
RSM: Response surface methodology
SDS–PAGE: Sodium dodecyl sulphate polyacrylamide gel electrophoresis
ANOVA: Analysis of variance
